# Analysis of ROQUIN, Tristetraprolin (TTP), and BDNF/miR-16/TTP regulatory axis in late onset Alzheimer’s disease

**DOI:** 10.3389/fnagi.2022.933019

**Published:** 2022-08-09

**Authors:** Mohammad Reza Asadi, Mahnaz Talebi, Jalal Gharesouran, Hani Sabaie, Abbas Jalaiei, Shahram Arsang-Jang, Mohammad Taheri, Arezou Sayad, Maryam Rezazadeh

**Affiliations:** ^1^Molecular Medicine Research Center, Tabriz University of Medical Sciences, Tabriz, Iran; ^2^Clinical Research Development Unit of Tabriz Valiasr Hospital, Tabriz University of Medical Sciences, Tabriz, Iran; ^3^Neurosciences Research Center (NSRC), Tabriz University of Medical Sciences, Tabriz, Iran; ^4^Cancer Gene Therapy Research Center, Zanjan University of Medical Sciences, Zanjan, Iran; ^5^Institute of Human Genetics, Jena University Hospital, Jena, Germany; ^6^Department of Medical Genetics, School of Medicine, Shahid Beheshti University of Medical Sciences, Tehran, Iran

**Keywords:** Alzheimer’s disease, TTP, ROQUIN, miR-16, BDNF, AU-rich elements, bioinformatics

## Abstract

Alzheimer’s disease (AD) is a heterogeneous degenerative disorder of the brain that is on the rise worldwide. One of the critical processes that might be disturbed in AD is gene expression regulation. Tristetraprolin (TTP) and RC3H1 gene (ROQUIN) are two RNA-binding proteins (RBPs) that target AU-rich elements (AREs) and constitutive decay elements (CDEs), respectively. TTP and ROQUIN, members of the CCCH zinc-finger protein family, have been demonstrated to fine-tune numerous inflammatory factors. In addition, miR-16 has distinct characteristics and may influence the target mRNA through the ARE site. Interestingly, BDNF mRNA has ARE sites in the 3’ untranslated region (UTR) and can be targeted by regulatory factors, such as TTP and miR-16 on MRE sequences, forming BDNF/miR-16/TTP regulatory axis. A number of two microarray datasets were downloaded, including information on mRNAs (GSE106241) and miRNAs (GSE157239) from individuals with AD and corresponding controls. R software was used to identify BDNF, TTP, ROQUIN, and miR-16 expression levels in temporal cortex (TC) tissue datasets. Q-PCR was also used to evaluate the expression of these regulatory factors and the expression of BDNF in the blood of 50 patients with AD and 50 controls. Bioinformatic evaluation showed that TTP and miR-16 overexpression might act as post-transcriptional regulatory factors to control BDNF expression in AD in TC samples. Instead, this expression pattern was not found in peripheral blood samples from patients with AD compared to normal controls. ROQUIN expression was increased in the peripheral blood of patients with AD. Hsa-miR-16-5p levels did not show significant differences in peripheral blood samples. Finally, it was shown that TTP and BDNF, based on evaluating the receiver operating characteristic (ROC), effectively identify patients with AD from healthy controls. This study could provide a new perspective on the molecular regulatory processes associated with AD pathogenic mechanisms linked to the BDNF growth factor, although further research is needed on the possible roles of these factors in AD.

## Introduction

Alzheimer’s disease (AD) is a progressive neurodegenerative disease that jeopardizes the physical and mental health of the elderly (Ding et al., 2022). The primary clinical signs of AD are memory loss and cognitive decline, which progress to dementia (Ding et al., 2022). It is estimated to affect 131.5 million people by 2050 if a certain cure is not found (Cummings et al., 2016). The global number of AD cases is estimated to be 35.6 million, with women accounting for a larger percentage than men (Prince et al., 2013). This massive number exerts an enormous strain on patients and their families (Wortmann, 2012). Despite tremendous attempts to treat the disease, AD remains incurable. It is believed that the significant failure rate of AD medication development is primarily attributable to our limited understanding of the disease’s complicated pathological process (Nguyen et al., 2021). There are several ideas about the primary origin of AD, illustrating the disease’s complexity. Cholinergic deficiency (Ferreira-Vieira et al., 2016), beta-amyloid toxicity (Aβ) (Lim et al., 2021), neurofibrillary tangles (NFTs) (Armstrong, 2009), tau protein hyperphosphorylation (Lewis and Dickson, 2016), neuroinflammation (Calsolaro and Edison, 2016), and dysregulation of neurotrophin growth factors (Amidfar et al., 2020) have all been indicated to be the risk factors for AD development. Regardless of the primary origin of AD, finding similar variables in different pathological pathways can aid in treating this complex disease.

Gene expression regulation is one of the primary mechanisms that can be affected in AD (Liang et al., 2008). In this regard, a large group of genes affects the production or clearance of Aβ, which are the part of the “AD genes” (Chen et al., 2013). Whereas it is well established that the expression levels of AD genes play a role in the etiology of AD, much regarding their particular regulation remains unclear (Ertekin-Taner, 2011). Thus, studying the regulatory components of disease genes and their related transcription factors is crucial to better understanding disease processes (Zhang et al., 2019). Post-transcriptional regulation is an effective form of gene expression regulation because it allows for the activation or repression of transcription and controls the number of proteins generated during translation (Zhang et al., 2019). Disruptions of normal transcriptional and post-transcriptional regulatory processes are increasingly recognized as causes of human disorders (Zhang et al., 2019). Studies have also been performed on post-transcriptional regulation in AD (Cui et al., 2018; Samadian et al., 2021; Song et al., 2021; Ghafouri-Fard et al., 2022). RNA-binding proteins (RBPs) and microRNAs (miRNAs) are among the most fundamental post-transcriptional regulation factors (Salerno et al., 2020), with the slightest alteration in the regulatory network imposed by RBPs and miRNAs leading to a larger-scale alteration in disease appearance (Carpenter et al., 2014). These factors act through specific sites on the 3′UTR of the target gene mRNA molecule. MiRNAs are small RNA molecules ranging in size from 19 to 25 nucleotides that govern the post-transcriptional silencing of target genes (Lu and Rothenberg, 2018). A single miRNA may target hundreds of mRNAs and alter the expression of several genes, many of which are engaged in functionally interacting pathways (Lu and Rothenberg, 2018). In general, a miRNA acts by binding to the microRNA response elements (MREs) in the 3′ untranslated region (3′UTR) and repressing mRNA translation by degradation or translational repression (Filipowicz et al., 2008). Similar to MREs, other sequences are found in 3′UTR of many mRNAs that can be targeted by certain RBPs, including the AU-rich elements (AREs), stem loop (SL), and constitutive decay elements (CDEs) (Caput et al., 1986; Hall, 2005; Glasmacher et al., 2010; Binas et al., 2020).

**FIGURE 1:**
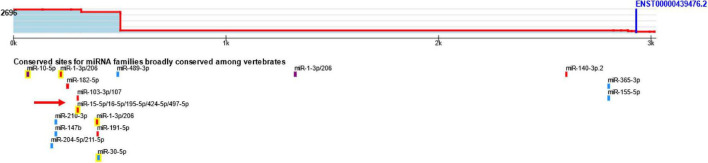
The predicted binding site of miR-16 on BDNF mRNA using TargetScan.

Tristetraprolin (TTP), RC3H1 gene (ROQUIN), and Regnase-1 are among the RBPs that target the AREs, CDEs, and SL sequences, respectively. TTP and ROQUIN have been shown to fine-tune the TNF-α and IL-6 inflammatory transcription mRNAs (Maeda and Akira, 2017) and belong to the CCCH zinc-finger protein family (Hall, 2005). Regulation of inflammatory cytokines is one of the potential roles played by TTP and ROQUIN, and in addition, studies have been performed on the dysregulation of these inflammatory factors in AD (Su et al., 2016). Interestingly, BDNF mRNA also has AREs in the 3′UTR and can be targeted by regulatory factors, such as TTP (Kumar et al., 2014). An increasing number of studies show that polymorphisms in the BDNF gene and reduced BDNF expression in the human brain are closely related to the pathogenesis of AD (Notaras and van den Buuse, 2019). Among these, C270T and Val66Met polymorphisms of the BDNF gene increase the susceptibility to AD (Fleitas et al., 2018). One of the prominent roles in post-transcriptional regulation is played by miRNAs with their specific functional positions, which are the MREs (Lu and Rothenberg, 2018). The miR-16 has unique features, and in addition to the MREs, it can exert its effect on the target mRNA through the ARE site (Calin et al., 2008). Overexpression of miR-16 is directly related to a decrease in the levels of inflammatory factors TNF-α and IL-6 and even affects TNF-α mRNA in addition to the MRE site through the ARE site (Yan et al., 2019). In addition, BDNF mRNA can be targeted by miR-16 according to bioinformatic studies through the MRE site (Figure 1) and according to the previous studies *via* ARE site (Bai et al., 2012). Given the importance of BDNF in the pathogenesis of AD, studies of the regulatory mechanisms of this growth factor are of particular importance. Since the effect of miR-16 and TTP, the BDNF/miR-16/TTP regulatory axis in AD can be considered. Therefore, the BDNF/miR-16/TTP regulatory axis in AD patients’ brain tissue and peripheral blood needs further investigation. AD is a brain disease, but pathogenic changes are not limited to the brain. In the recent years, studies have shown that changes in the blood are also detectable, and peripheral blood contains essential information for detecting pathogenic and physiological changes, so analyzing the gene expression of blood samples suggests an opportunity to study AD-related changes non-invasively with no need for brain biopsy and brain-spinal fluid examinations that are challenging (Caput et al., 1986; Sharp et al., 2006; Booij et al., 2011; Zou et al., 2020).

The purpose of this study was to determine the levels of TTP and ROQUIN expression in AD brain tissues, as well as the BDNF/miR-16/TTP regulatory axis and their use as peripheral factors related to the development of AD using bioinformatic and experimental methods.

## Materials and methods

### Regulatory axis design

The genes that impact BDNF through ARE sites were found using the ARE site database (Fallmann et al., 2016), a database for AREs, and direct evidence for interaction and lifetime regulation through choosing the ATTTA motif and inserting ENSG00000176697 as the target gene (BDNF). To find a miRNA to study, BDNF was entered as a target gene into the miRTarBase (Huang et al., 2020) and miRWalk (version 3) (Sticht et al., 2018) databases. These two databases are among the databases that provide validated miRNA-target interactions. TargetScan was used to visualize the binding sites of miRNAs to BDNF.

### Bioinformatic analysis using brain microarray dataset

#### Data collection on gene expression profiles

The purpose of this study was to mine data from a microarray dataset of human temporal cortex (TC) tissue samples with varying degrees of AD-related neurofibrillary pathology using a bioinformatic technique (GSE106241). This study aimed to determine the changes in the expression of ROQUIN, TTP, and BDNF in human TC tissue samples. The above gene expression profile was retrieved from the NCBI Gene Expression Omnibus database (GEO) (Barrett et al., 2013). A chip-based platform, GPL24170, Agilent-044312, Human 8 × 60K Custom Exon array (Probe Name version), was used for the dataset. GSE106241 includes 60 human TC tissue samples divided into seven groups according to Braak staging (Braak 0: *n* = 6, Braak I: *n* = 11, Braak II: *n* = 11, Braak III: *n* = 6, Braak IV: *n* = 7, Braak V: *n* = 12, Braak VI: *n* = 7) (Marttinen et al., 2019). We evaluated the expression of these genes during stages III through VI, which corresponds to the disease’s most severe form. Braak staging is a method of neuropathological staging that differentiates among early, medium, and advanced AD based on the development of NFTs within the medial temporal lobe memory circuit. Braak stage 0 correlates with the lack of NFTs, stages I–II to NFTs in the entorhinal–perirhinal cortex, stages III–IV to NFTs in the hippocampus as well, and stages V–VI to NFTs scattered across the neocortical areas (Braak and Braak, 1991).

#### Data preprocessing and the identification of differentially expressed genes

Background correction (Silver et al., 2009) was conducted with an offset of 15 using the normexp technique, and between-array normalization was performed using the quantile approach using the normalizeBetweenArrays () function from the linear models for microarray data (limma) R package (Ritchie et al., 2015). Only spots with signal minus background flagged as “positive and significant” (field name gIsPosAndSignif) and not flagged as ControlType or IsManualFlag were used. The AgiMicroRna Bioconductor package (version 2.40.0) was utilized to assess the quality. Using the linear models for microarray data (limma) R package (Ritchie et al., 2015) in Bioconductor (Huber et al., 2015), differentially expressed gene analysis (DEGA) was done to compare AD samples to normal samples. Student’s *t*-test was performed to determine whether genes were statistically significant. Additionally, the following cutoff values for aberrantly expressed mRNAs were used: a false discovery rate (adjusted *p*-value) < 0.001 (Kang et al., 2020), and |log_2_ fold change (log_2_FC)| > 0.5 (Fan et al., 2020). The volcano plot for DEGs and the heatmap for ROQUIN, TTP, and BDNF genes were created using the R packages Enhanced Volcano (version 1.8.0) and Pheatmap (version 1.0.12).

#### Identification of miR-16 expression levels associated with Alzheimer’s disease and prediction of its interactions with brain-derived neurotrophic factor

We used the same bioinformatic approach mentioned before to identify the differentially expressed miRNAs (DEmiRNAs) associated with AD pathogenesis. The NCBI GEO database was used to obtain data on the miRNA profile of GSE157239. The platform GPL21572 (miRNA-4) Affymetrix Multispecies miRNA-4 Array (ProbeSet ID version) was used for the dataset. The GSE157239 study included eight TC samples from patients with AD (Braak stage III or above) and eight TC samples from healthy controls. The Robust Multichip Average (RMA), a powerful tool included in the affy Bioconductor package, was used to compensate for background and normalize the quantiles of all raw data files (Irizarry et al., 2003). Interquartile range filtering (IQR across samples on the log base two-scale greater than the median IQR) was used to reduce the number of analyzed genes, followed by an intensity filtering (a minimum of > 100 expression signals in a minimum of 25% of the arrays) to eliminate insignificant probe sets that are not expressed or changing (von Heydebreck et al., 2004). The AgiMicroRna Bioconductor package was used to determine the quality. The DEGA was performed on normal and AD samples in Bioconductor (Huber et al., 2015) using the limma R package (Ritchie et al., 2015). The miRNAmeConverter Bioconductor package (Haunsberger et al., 2017) converted the miRNA names to miRbase v22. Student’s *t*-test was performed to determine whether miRNAs were statistically significant. Additionally, the following cutoff values for aberrantly expressed miRNAs were used: a false discovery rate (adjusted *p*-value) < 0.001 (Kang et al., 2020), and |log_2_ fold change (log_2_FC)| > 0.1 (Fan et al., 2020). The DEmiRNA heatmap was built using the R package Pheatmap. Finally, miR-16 expression levels were examined from the data. The interaction between miR-16 and BDNF mRNA was identified using miRWalk (version 3) (Sticht et al., 2018) and miRTarBase (Huang et al., 2020).

#### Correlation analysis among ROQUIN, tristetraprolin, and brain-derived neurotrophic factor

Pearson’s correlation analysis was used to assess whether there were any positive correlations in the regulatory axis among ROQUIN, TTP, and BDNF. Correlations and visualizations were calculated and visualized using the Hmisc and psych packages.

### Identification and differential analysis of tristetraprolin and ROQUIN expression levels and the brain-derived neurotrophic factor/miR-16/tristetraprolin regulatory axis in peripheral blood

#### Participants and peripheral blood samples

This case-control study comprised of 100 participants, 50 patients with sporadic AD, and 50 age- and gender-matched healthy controls. The clinical research ethics committee of Tabriz University of Medical Sciences approved this study (ethical code: IR.TBZMED.REC.1398.1263). Individuals from Tabriz University of Medical Sciences’ Imam Reza Hospital were recruited to participate in this study. A neurology specialist used the DSM-V criteria (Vahia, 2013) to identify the individuals. In selecting the cases, the patients’ medical records were reviewed, including the disease’s duration, education level, family history, history of other diseases, and their duration, the drugs used, and the results of the Mini-Mental State Examination. Patients who met the inclusion criteria were selected for sampling. The inclusion criteria were that one was 65 years of age or older and had no other psychiatric or neurological diagnosis other than AD. The control group consisted of individuals without AD in the same department who were 65 years old or older. Based on the exclusion criteria, diabetes, active or chronic infectious illnesses, thyroid disorders, cancer, renal and liver failure, inflammatory diseases or use of anti-inflammatory medications, metabolic disease, severe ischemic heart disease, alcohol misuse, cerebrovascular accident, and having taken corticosteroids during the preceding 8 weeks of examination were all excluded. Cognitive function was assessed using MMSE in both groups. Before joining the study, all individuals or their principal caregivers provided written informed permission. Finally, 5 ml of peripheral blood was collected from each participant and placed in EDTA-treated tubes.

#### Expression assays

Total RNAs were obtained from whole blood using the Hybrid-R™ Blood RNA purification kit (GeneALL, Seoul, South Korea) and then processed with DNase I to eliminate DNA contamination. NanoDrop was used to determine the quantity and quality of isolated RNA (Thermo Scientific, Wilmington, DE, United States). The cDNA synthesis kit (GeneALL) was used to produce complementary DNA (cDNA) in accordance with the manufacturer’s instructions. The cDNA was preserved at 20°C for additional investigation. Table 1 lists the primer sequences used in reverse transcription and quantitative polymerase chain reaction (qPCR) experiments. To normalize miRNA and mRNA levels, internal controls, such as U6 and ubiquitin C (UBC), were used. The qPCR was performed using the Step OnePlus™ Real-Time PCR and the RealQ Plus2x Master Mix (Ampliqon, Odense, Denmark). All qPCRs were accomplished in duplicate.

**TABLE 1:** List of primers used in this study.

Gene name	Primer sequences (5 ′–3′)	Product size	Tm
*TTP*	Forward primer TCTGACTGCCATCTACGAGAGCC Reverse primer TCAGTCTTGTAGCGCGAGGG	315 nt	61
*ROQUIN*	Forward primer AGTGCTAGAGGAGTGGGTCTG Reverse primer ATCCCCTAGCCCTTACTGCTG	232 nt	60.5
*BDNF*	Forward primer GGAAAACTTGGGAGGCGGAAT Reverse primer TCTCACCTGGTGGAACCATT	234 nt	59
*UBC*	Forward primer CAGCCGGGATTTGGGTCG Reverse primer CACGAAGATCTGCATTGTCAAGT	72 nt	60
*hsa-miR-16-5p*	RT primer GTCGTATCCAGTGCAGGGTCCGAGGT ATTCGCACTGGATACGACCGCCAA Forward primer CAGCCGGGATTTGGGTCG Reverse primer CACGAAGATCTGCATTGTCAAGT	−	60
*U6*	RT primer GTCGTATCCAGTGCAGGGTCCGAGGTATTCGC ACTGGATACGACAAAAATAT Forward primer GCTTCGGCAGCACATATACTAAAAT Reverse primer CGCTTCACGAATTTGCGTGTCAT	−	60

#### Quantitative polymerase chain reaction statistical analysis

The R v.4 software packages brms, stan, pROC, and GGally were used to analyze the data. The Bayesian regression model was used to evaluate the relative expressions of BDNF, TTP, ROQUIN, and hsa-miR-16-5p in patients with AD, healthy controls, and subgroups. The effects of age and gender have been corrected. Adjusted *p*-values of less than 0.05 were considered significant. The genes, as mentioned earlier expression was also examined across age groups and between men and women. The Spearman correlation coefficients were used to assess the connections between the variables in the study. The diagnostic power of the genes was assessed by utilizing a receiver operating characteristic (ROC) curve study.

## Results

### Identification of brain-derived neurotrophic factor/miR-16/tristetraprolin regulatory axis

The results obtained from ARE site database indicated that TTP targets BDNF through ARE sites. In addition, hsa-mir-16-5p was identified by miRTarBase and miRWalk databases as one of the miRNAs capable of targeting BDNF. Figure 1 illustrates the binding site of miR-16 on the BDNF mRNA using the TargetScan database.

### Reanalysis of brain microarray datasets

#### Identification of differentially expressed genes

Before DEGA, background correction, batch modification, gene filtering, and normalization were all carried out. To assess the quality, the AgiMicroRna Bioconductor package was used. Box plots from the gene expression dataset were used to assess data distribution after normalization. The box plots showed that separate arrays in the box plots had identical expression level medians, indicating that the adjustment was made correctly.

**FIGURE 2:**
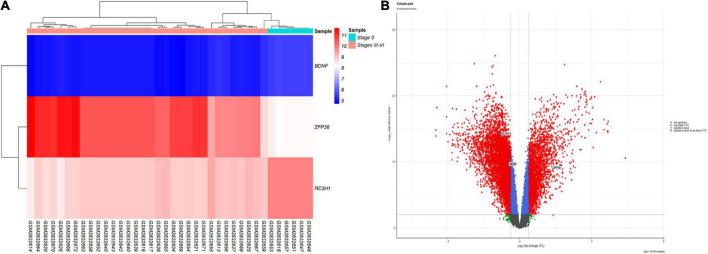
Differentially expressed mRNAs in brain samples from patients with AD in Braak stages III to VI and control (CTL) samples. **(A)** Gene-specific heatmaps for the BDNF, TTP, and ROQUIN genes. Genes with a high level of expression are depicted in red, whereas those with a low level of expression are displayed in blue. **(B)** The DEGs’ volcano plan. Screening for DEGs was done using a | (log_2_FC) | > 0.5 and an adjusted *p*-value 0.001.

According to the results, TTP was significantly upregulated (log_2_FC = 2.246849, adj.P.Val = 2.97E–10), whereas BDNF (log_2_FC = −0.54698, adj.P.Val = 5.33E–10) and ROQUIN (log_2_FC = −0.70881, adj.P.Val = 1.36E–14) were significantly downregulated in Braak stages III–IV. Table 2 provides details of above-mentioned genes expression levels in Braak stage III–IV. The criteria for the identification of DEGs were adjusted *p*-value < 0.001, and | log_2_ fold change (log_2_FC) | > 0.5. Figure 2 depicts a volcano plot of DEGs, as well as a hierarchical clustering heatmap of BDNF, ROQUIN, and TTP genes in Braak stages III to IV.

**TABLE 2:** Expression levels of TTP, RC3H1, BDNF, and miR-16 genes in TC tissue samples.

Gene symbol	Log_2_FC	Average expression	*t*-test	*P*-value	adj.P.-val	B-statistic
TTP	2.246849	10.15882	8.950171	4.50E-11	2.97E−10	14.87444
BDNF	−0.54698	5.117175	−8.73636	8.59E−11	5.33E−10	14.22688
RC3H1	−0.70881	9.071129	−13.155	4.54E−16	1.36E−14	26.43185
hsa-miR-16-5p	0.170641094	10.40612988	6.4211615	1.20E−05	2.24E−05	0.775078547

#### Identification of miR-16 expression levels associated with Alzheimer’s disease

**FIGURE 3:**
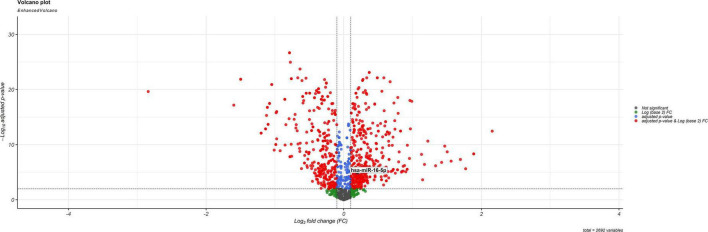
Volcano plot for the DEmiRs. The DEmiRs were screened based on a | (log_2_FC) | > 0.1 and an adjusted *p*-value < 0.001.

Gene filtering, normalization, batch adjustment, and background correction were conducted before DEGA. Quality was monitored using the AgiMicroRna Bioconductor package. After normalization, box plots for gene expression profiles were provided to assess the data distribution. The box plots’ expression level medians of separate arrays were comparable, indicating a proper adjustment. MiR-16 has been upregulated (log_2_FC = 0.170641094, adj.P.Val = 2.24E–05) according to the selected criteria: adjusted *p*-value < 0.001, and | log_2_ fold change (log_2_FC) | > 0.1 among DEmiRNAs. Figure 3 illustrates a DEmiRNA volcano plot. Table 2 summarizes the details concerning the miR-16 expression level.

#### Correlation analysis among ROQUIN, tristetraprolin, and brain-derived neurotrophic factor

**FIGURE 4:**
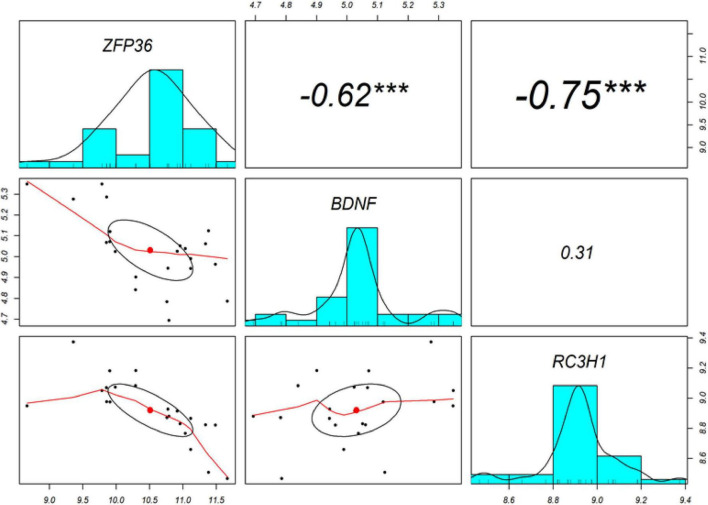
The diagonal depicts the distribution of each variable. The bottom section of the diagonal depicts bivariate scatter plots with a fitted line. The correlation coefficients and significance level are presented as stars on the top section of the diagonal. ***Indicates a significant association with a *p*-value < 0.001.

The Pearson’s correlation analysis was performed among ROQUIN, TTP, and BDNF to confirm the regulatory axis hypothesis, which indicates that TTP and miR-16 regulate BDNF. Significant negative correlations were identified between the levels of expression of BDNF-TTP (*r* = −0.62, *p* < 0.001) and ROQUIN-TTP (*r* = −0.75, *p* < 0.001) (Figure 4).

### Identification and differential analysis of tristetraprolin and ROQUIN expression levels and the brain-derived neurotrophic factor/miR-16/tristetraprolin regulatory axis in peripheral blood

#### General demographic data

Following assessment of the inclusion and exclusion criteria, the study comprised of 50 patients with AD (male/female%: 31.4/68.6) with age [mean standard ± deviation (SD)] of 76.36 ± 6.26 and 50 healthy controls (male/female%: 30.6/69.4) with age (mean ± *SD*) of 74.3 ± 6.22. The patient and control groups’ MMSE scores (mean ± *SD*) were 14.2 ± 5.94 and 27.5 ± 0.76, respectively.

#### Expression assays

**FIGURE 5:**
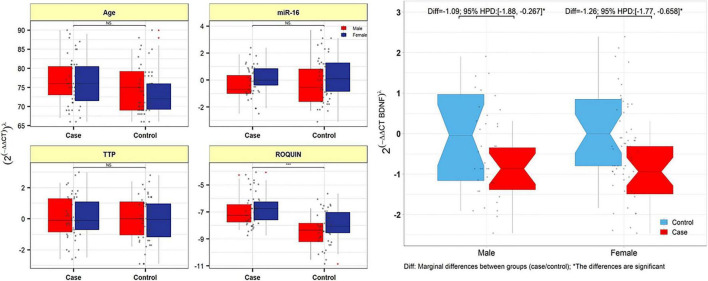
TTP, ROQUIN, hsa-miR-16-5p, and BDNF expression in peripheral blood samples from patients and controls. Gray dots show values. The means of expression levels and the interquartile range are shown.

The relative expression levels of the genes BDNF, ROQUIN, TTP, and hsa-miR-16-5p in patients with AD and controls are shown in Figure 5. TTP expression levels in PB samples showed no significant differences between patients with AD and healthy controls (adjusted *p*-value = 0.949), nor between subgroups. BDNF expression was considerably reduced in PB samples from patients with AD compared to controls (posterior beta = −1.066, adjusted *p*-value = 0.005). Male and female subgroups showed similar decreased expression (posterior beta = −1.092, adjusted *p*-value = 0.048 and posterior beta = −1.257, adjusted *p*-value = 0.007, respectively). ROQUIN expression was considerably higher in PB samples from patients with AD than in controls (posterior beta = 0.858, adjusted *p*-value = 0.013). The male and female subgroups also showed increased expression (posterior beta = 0.812, adjusted *p*-value = 0.046 and posterior beta = 0.811, adjusted *p*-value = 0.002, respectively). There were no significant differences in hsa-miR-16-5p expression in PB samples from patients with AD and healthy controls (adjusted *p*-value = 0.701), as well as subgroups. Tables 3–6 provide detailed data on the relative expressions of BDNF, TTP, ROQUIN, and hsa-miR-16-5p, respectively.

**TABLE 3:** Relative levels of BDNF in AD cases and controls according to the Bayesian quantile regression model.

	*BDNF*	Posterior Beta of [2^(–ddct)^]^ʎ^	SE	Adjusted *P*-value*	95% Crl for beta
Total	Group, case vs. control	–1.066	0.416	0.005	[−1.818, −0.197]
	Sex, female vs. male	0.078	0.364	0.835	[−0.598, 0.847]
	Age (years)	0.004	0.01	>0.999	[−0.014, 0.023]
	Group * sex	–0.187	0.468	0.491	[−1.091, 0.708]
Male	Case vs. control	–1.092	−0.267	0.048	[−0.267, −1.881]
Female	Case vs. control	–1.257	−0.658	0.007	[−0.658, −1.77]

*Estimated from frequentist methods; CrI, credible interval; ʎ, power transformation value estimated from Box-cox or Yeo-Johnson method.

**TABLE 4:** Relative levels of TTP in AD cases and controls according to the Bayesian quantile regression model.

	TTP	Posterior Beta of [2^(–ddct)^]^ʎ^	SE	Adjusted *P*-value*	95% Crl for beta
Total	Group, case vs. control	**0.07**	**0.33**	0.949	**[**−**0.61, 0.71]**
	Sex, female vs. male	**0.02**	**0.27**	0.837	**[**−**0.5, 0.58]**
	Age (years)	−**0.019**	**0.02**	0.241	**[**−**0.05, 0.01]**
	Group * sex	−**0.084**	**0.43**	0.96	**[**−**0.91, 0.74]**
Male	Case vs. control	**0.037**	**0.23**	0.814	**[**−**0.42, 0.48]**
	Age	−**0.019**	**0.01**	0.424	**[**−**0.05, 0.01]**
Female	Case vs. control	**0.036**	**0.23**	0.965	**[**−**0.45, 0.47]**
	Age	−**0.019**	**0.01**	0.810	**[**−**0.05, 0.01]**

*Estimated from frequentist methods; CrI, credible interval, ʎ, power transformation value estimated from Box-cox or Yeo-Johnson method. The bolded values indicate the significance of that data among the rest of the data.

**TABLE 5:** Relative levels of ROQUIN in AD cases and controls according to the Bayesian quantile regression model.

	*ROQUIN*	Posterior Beta of [2^(–ddct)^]^ʎ^	SE	Adjusted *P*-value*	95% Crl for beta
Total	Group, case vs. control	**0.858**	**0.26**	0.013	**[0.38, 1.38]**
	Sex, female vs. male	−**0.177**	**0.22**	0.432	**[**−**0.61, 0.27]**
	Age (years)	**0.008**	**0.01**	0.564	**[**−**0.02, 0.03]**
	Group * sex	−**0.043**	**0.32**	0.961	**[**−**0.67, 0.56]**
Male	Case vs. control	**0.812**	**0.16**	**0.046**	**[0.51, 1.13]**
	Age	**0.011**	**0.01**	0.317	**[**−**0.02, 0.04]**
Female	Case vs. control	**0.811**	**0.15**	**0.002**	**[0.51, 1.12]**
	Age	**0.01**	**0.01**	0.852	**[**−**0.02, 0.03]**

*Estimated from frequentist methods; CrI, credible interval, ʎ, power transformation value estimated from Box-cox or Yeo-Johnson method. The bolded values indicate the significance of that data among the rest of the data.

**TABLE 6:** Relative levels of miR-16 in AD cases and controls according to the Bayesian quantile regression model.

	hsa-miR-16-5p	Posterior Beta of [2^(–ddct)^]^ʎ^	SE	Adjusted *P*-value*	95% Crl for beta
Total	Group, case vs. control	**0.24**	**0.28**	0.701	**[**−**0.38, 0.75]**
	Sex, female vs. male	−**0.455**	**0.2**	0.51	**[**−**0.84,** -**0.04]**
	Age (years)	−**0.004**	**0.01**	0.693	**[**−**0.03, 0.02]**
	Group * sex	−**0.054**	**0.34**	0.94	**[**−**0.7, 0.62]**
Male	Case vs. control	**0.147**	**0.21**	0.727	**[**−**0.32, 0.54]**
	Age	−**0.0002**	**0.02**	0.447	**[**−**0.03, 0.03]**
Female	Case vs. control	**0.137**	**0.22**	0.955	**[**−**0.32, 0.54]**
	Age	−**0.0003**	**0.02**	0.607	**[**−**0.03, 0.03]**

The bolded values indicate the significance of that data among the rest of the data.

#### Correlation analysis

**FIGURE 6:**
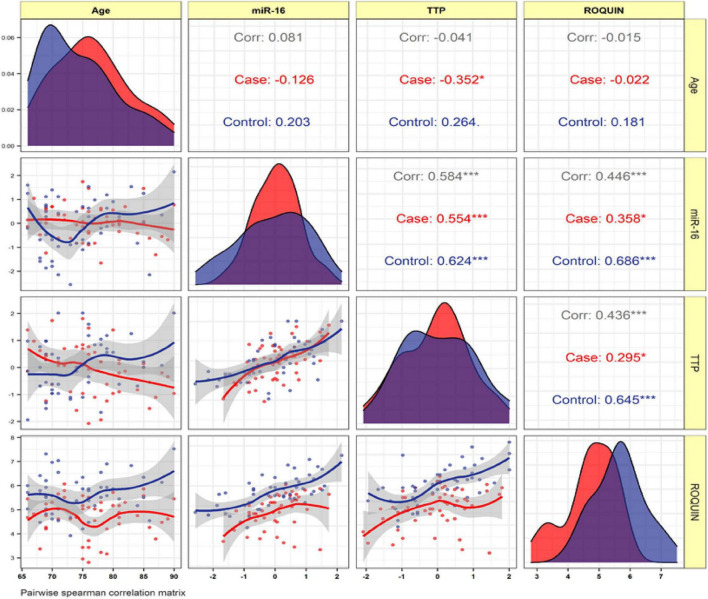
The variable distribution is displayed on the diagonal. The correlation coefficients, as well as the significance level, are shown as stars. * and ***represent a significant correlation at *p* < 0.05 and *P* < 0.001, respectively.

Expression levels of ROQUIN, TTP and hsa-miR-16-5p were not correlated with the age of patients. In patients with AD and control samples, TTP expression was positively correlated with miR-16 (*r* = 0.584, *p* < 0.001). Positive correlation between ROQUIN and miR-16 expression (*r* = 0.446, *p* < 0.001) and ROQUIN and TTP (*r* = 0.436, *p* < 0.001) with more significant values in control samples than patients was seen (Figure 6).

#### Receiver operating characteristic curve analysis

**FIGURE 7:**
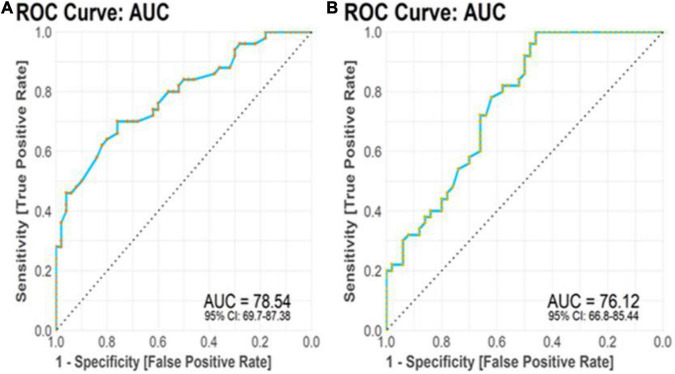
Receiver operating characteristic (ROC) curve analysis. **(A)** TTP transcript levels displayed diagnostic power of 0.7854. **(B)** BDNF transcript levels displayed diagnostic power of 0.7612.

The TTP and BDNF were evaluated for their diagnostic utility in differentiating patients with AD from controls. We achieved significant diagnostic powers of 0.7854 (*SE* = 87.38, *SP* = 69.7) and 0.7612 (*SE* = 85.44, *SP* = 66.8) from the transcript levels of TTP and BDNF, respectively, by measuring the area under the curve (AUC) (Figure 7).

## Discussion

So far, many studies have been conducted on the ARE sites and their potential effects on regulating gene expression in other diseases, including cancer, and confirm that this mechanism of regulating gene expression can cause dysregulation in target genes (reviewed in Khabar, 2017). Among these, gene expression regulation in the CNS, and more specifically in the brain, has the most complex and, at the same time, the most precise regulation, and in the case of dysregulation, it shows it results in various forms of diseases (reviewed in Goldie and Cairns, 2012). Many studies have been conducted on the gene expression regulation mechanisms in AD as one of the major neurodegenerative diseases (NDD) from the past to the present (reviewed in Goldie and Cairns, 2012; Ułamek-Kozioł et al., 2016). There have even been studies of other members of the RNA-binding protein family targeting this ARE site in AD and other NDDs, but their potential role in this site has not been addressed (LaJevic et al., 2010; Mansfield and Keene, 2012). Identifying this regulatory mechanism could explain related pathogenic processes and offer new treatment options. Our efforts to understand the various aspects of regulatory processes of gene expression in AD pathogenesis clarify possible molecular targets, identifying factors involved in the disease developing based on the exact regulatory mechanism.

### Tristetraprolin and ROQUIN as a member of the CCCH-type zinc-finger protein family in Alzheimer’s disease

The results of the bioinformatic evaluation showed that, as expected, ROQUIN expression showed a significant decrease in TC samples compared to healthy controls. On the other hand, TTP has an increased expression in TC samples compared to healthy controls. So far, studies on other types of zinc-finger family proteins (C2H2 type, MYM type, ZC3H14 type, and Matrin type) have been performed in AD (reviewed in Bu et al., 2021). Meanwhile, MSUT2 (ZC3H14), belonging to the CCCH family, ultimately affects the accumulation of tau proteins to affect NFTs (Baker et al., 2020). MSUT2, also known as the mammalian suppressor of tau pathology-2, binds to the polyadenosine tail (poly[A]-tail) of mRNAs through the zinc-finger domain CCCH-type at its C-terminal, leading to the formation of pathological tau protein (Baker et al., 2020). Tau proteins are a collection of six protein isoforms generated by alternative splicing of the microtubule-associated protein tau (MAPT) gene, and their major role is to preserve the integrity of microtubules in axons owing to their high expression in CNS neurons (Asadi et al., 2021). In addition, loss of MSUT2 function can reduce tau protein formation and prevent neuronal death, but the molecular mechanism is still unclear, and the only conjecture that can be made is the shared function of MSUT2 and PABPN1 (Baker et al., 2020). Other studies have shown that MSUT2 knockout has anti-inflammatory and neuroprotective effects in the mouse model (Wheeler et al., 2019). ROQUIN and TTP are the primary genes that regulate inflammatory factors. A significant decrease in ROQUIN expression in TC samples indicates an increase in inflammatory factors and thus an increase in inflammation in TC tissue. By acting on the CDE sequences and utilizing Ccr4 and Caf-1 factors, ROQUIN destabilizes the target gene mRNA molecule and reduces its expression (Leppek et al., 2013). Among the significant inflammatory factors with CDE sequence are TNF-α and IL-6 (Zhao et al., 2008), ROQUIN is also responsible for regulating the expression of co-stimulatory receptor molecules, such as Icos, CTLA-4, and Ox40 (Tavernier et al., 2019). On the other hand, the point to be noted is the increased expression of ROQUIN in the peripheral blood of patients with AD compared to healthy controls. Studies on inflammation and expression of inflammatory factors TNF-α and IL-6 in AD indicate an increase in the expression of these factors in the peripheral blood and even mention the increase in these factors as a biomarker in AD (Kim et al., 2017).

However, increased ROQUIN expression in the peripheral blood of patients with AD may indicate that ROQUIN is not involved in the mechanism of regulating the expression of inflammatory factors in AD. A recently published study raises the possibility that ROQUIN regulates the expression of silent mRNAs (Mino et al., 2015). IL-2 is one of the main inflammatory factors affected by the regulation of ROQUIN by acting on the CDE sequence (Kim et al., 2012). IL-2, along with TNF-α and IL-6, plays a significant role in regulating the profile of immune cells (Paul-Samojedny et al., 2013). Since patients with AD have their immune cell profiles (Frost et al., 2019), increased ROQUIN expression may be one of the levers of this disease in regulating the number and characteristics of immune cells in the peripheral blood of patients with AD, which has not been addressed in this study and could be the basis for further studies.

The increase in TTP expression obtained by bioinformatic studies in the TC tissue sample of patients with AD compared to healthy controls indicates that TTP does not play an essential role in regulating the expression of inflammatory genes in the brain. However, this increase should not be underestimated, as TTP is not the only regulator of inflammatory gene expression and has the potential to regulate gene expression by ARE sites, each of which may play an essential role in the pathogenesis of AD. In this regard, no significant difference in TTP expression levels in the peripheral blood of patients with AD compared to healthy controls indicates the lack of a role for the TTP gene in the mechanisms of gene expression regulation in the peripheral blood.

### Brain-derived neurotrophic factor as a target for tristetraprolin and miR-16 in Alzheimer’s disease

Brain-derived neurotrophic factor, the most diffuse neurotrophin in the central nervous system, plays a crucial role in synaptic plasticity and neuronal survival (Leal et al., 2017). Accumulated evidence suggests that BDNF polymorphisms and decreased BDNF expression in the human brain are closely related to the pathogenesis of AD (Leal et al., 2015). It has been suggested that C270T and Val66Met polymorphisms of the BDNF gene increase the ADs susceptibility and that individuals with AD have decreased levels of BDNF mRNA and protein in serum and brain compared with healthy elderly (Olin et al., 2005; Tsai, 2018). Significantly, higher BDNF expression reduces cognitive impairment in the elderly. In addition, advanced neuropathology of AD suggests that BDNF levels in the brain can be used as a new marker to assess the progression of AD (Mori et al., 2021). *In vitro* and *in vivo* experiments show that BDNF has neuroprotective effects against the cytotoxic effects of beta-amyloid plaques and Aβ-induced learning disabilities (Jiao et al., 2016). In this regard, BDNF gene delivery in transgenic mice promotes Aβ precursor protein (APP), improves learning and memory, increases synapse protection, and reduces APP mutation cell loss (Psotta et al., 2015). These lines of evidence suggest that direct use of BDNF exerts a protective effect against Aβ-related pathologies.

Brain-derived neurotrophic factor microRNA was identified as the target of TTP by the database for AREs and direct evidence for interaction and lifetime regulation (Fallmann et al., 2016). On the other hand, miRTarBase and miRWalk databases were used to identify microRNAs with a common purpose of BDNF (Sticht et al., 2018; Huang et al., 2020). Among these, hsa-miR-16 was selected as the desired microRNA. MiR-16 is one of the few microRNAs capable of acting on AREs (Jing et al., 2005). Thus, an expression regulatory axis related to BDNF is created through two ARE sites that TTP and miR-16 target and the MRE site, which is targeted by miR-16, where this regulation occurs. Remarkably, miR-16 is one of the prominent miRNAs in inflammatory pathways by targeting inflammatory factors, such as TNF-α, IL-6, IL-4, and IL-8 (reviewed in Yan et al., 2019). Our bioinformatic analysis demonstrated that BDNF expression significantly decreased (log_2_FC = −0.54698, adj.P.Val = 5.33E–10) in TC tissues compared to healthy controls, TTP expression had increased significantly (log_2_FC = 2.246849, adj.P.Val = 2.97E–10), and miR-16 expression also increased (log_2_FC = 0.170641094, adj.P.Val = 2.24E–05) in TC tissues. Brain tissue has its expression profile and transcript complexity compared to other tissues (Naumova et al., 2013). The amounts of changes obtained from bioinformatic analysis, except for TTP, do not exceed 2-folds. However, it should be noted that these factors are among the influential factors, namely, RBPs and miRs, in regulatory gene expression networks; the slightest changes in their expression can be followed in disease manifestation. Expression correlation studies, as a result, showed a negative correlation (*r* = −0.62, *p* < 0.001) between TTP and BDNF in TC tissues in AD. Gene correlation analysis attempts to categorize human genes by considering their co-expression levels (Michalopoulos et al., 2012).

On the one hand, Kumar et al. introduced TTP as a regulator of BDNF in the C2C12 cell line, a derivative of myoblast cells (Kumar et al., 2014). By acting on the ARE site and through the use of destabilizing factors, TTP causes instability of the target mRNA molecule and reduces its expression. On the other hand, miR-16 and miR-15 were among the first microRNAs implicated in chronic lymphocytic leukemia (Calin et al., 2002). MiR-16 may remove or inhibit the translation of this neurotrophic factor by acting on the RISC complex structure and acting on the MRE site of BDNF mRNA (Figure 1). It should be noted that miR-16 contains a UAAAUAUU sequence that complements the ARE site, which was one of the reasons for its selection among other miRs (Brooks and Blackshear, 2013). There is an indirect collaboration between TTP and miR-16 through the RISK complex, and miR-16 has the ability to accompany TTP in targeting mRNAs containing ARE sites (Jing et al., 2005).

The results of studies of TTP (adjusted *p*-value = 0.949) and miR-16 (adjusted *p*-value = 0.701) gene expression in the peripheral blood of patients with AD compared to healthy controls did not support this hypothesis, nor did the results of bioinformatic analysis. However, the expression of BDNF in the peripheral blood of patients with AD has experienced a significant decrease, although the expression correlation analysis of TTP and miR-16 is positive (*r* = 0.584, *p* < 0.001) and indicates an expression relationship between these two factors. Positive expressive correlation, although not very high value, and the cooperation between TTP and miR-16 can be considered in one direction; however, a weak correlation may not even indicate a direct impact. Given the ROC value of the TTP gene, it can be identified as one of the genes that may have diagnostic value in AD. Decreased expression of BDNF in both brain and peripheral blood is a common feature of patients with AD, and further attention and studies in this field can work to eliminate one of the levers of this disease.

## Limitations

Our research had some limitations. First, various aspects, such as different methods, sample preparation, platforms, data analysis, and patient’s characteristics, may affect gene expression patterns. Second, the limited number of samples may lead to a lack of statistical validity. In addition, our bioinformatic analysis must be validated by practical techniques. Third, this expression regulation mechanism is best investigated at the protein level and with related techniques, such as ELISA and Western blotting. Finally, we did not examine the expression of TTP, ROQUIN, and hsa-miR-16-5p in peripheral blood cell subpopulations.

## Conclusion

The genes encoding the CCCH-type zinc-finger protein family play the essential roles in the underlying mechanisms in many cells. These roles, including the most important ones, such as regulating gene expression, create the potential for this gene family, which may cause disease and pathogenesis in a particular complication. Learning more about these genes and the regulatory axes involved can help to explain pathogenic processes and provide opportunities for new therapies. As a result, our efforts to understand the various aspects of regulatory processes through the family of CCCH-type zinc-finger proteins in AD pathogenesis led to new insights into possible molecular targets and to the discovery of biomarkers based on these regulatory pathways. To the best of our knowledge, this study is the first evidence of one of these regulatory axes implemented by TTP and ROQUIN in AD patients’ peripheral brain and blood. The effect of TTP and miR-16 on the BDNF expression regulation was also investigated as one of the aspects of this regulation due to its effect on the ARE site. The results are preliminary, and further, *in vitro* and *in vivo* studies may reinforce these findings. Whereas the possible role of these regulatory axes at the transcript level requires further exploration, this study develops insights into one of the regulatory aspects of critical and influential factors at the transcript level. It provides a new perspective on the molecular processes of AD development.

## Data availability statement

The original contributions presented in the study are publicly available. This data can be found here: https://www.ncbi.nlm.nih.gov/geo/query/acc.cgi?acc=GSE106241 and https://www.ncbi.nlm.nih.gov/geo/query/acc.cgi?acc=GSE157239.

## Ethics statement

The clinical research Ethics Committee of Tabriz University of Medical Sciences approved the current study (Ethical code: IR.TBZMED.REC.1398.1263). The patients/participants provided their written informed consent to participate in this study.

## Author contributions

MA, HS, MAT, and AJ performed the experiment and collected the samples. SA-J and AS analyzed the data. MT and JG designed and supervised the study. MR and MA wrote the draft and revised it. All authors read and confirmed the submitted version.

## Abbreviations

AD: Alzheimer’s disease
TTP: Tristetraprolin
RBP: RNA-binding proteins
MRE: microRNA response element
ARE: AU-rich elements
CDEs: constitutive decay elements
UTR: untranslated region
BDNF: brain-derived neurotrophic factor
TC: temporal cortex
ROC: receiver operating characteristic
NFT: neurofibrillary tangle
miRNA: microRNA
SL: stem loop
GEO: Gene Expression Omnibus database
DEG: differentially expressed genes
DEGA: differentially expressed gene analysis
DEmiRNA: differentially expressed miRNA
RMA: Robust Multichip Average
MMSE: Mini-Mental State Examination
UBC: ubiquitin C
SD: standard deviation
AUC: area under the curve
CNS: central nervous system
NDD: neurodegenerative disease
MSUT2: mammalian suppressor of tau pathology-2
SE: sensitivity
SP: specificity
